# In Situ Calibration of Terahertz Time-Domain Polarimetry Systems with a Leaky Wire Grid Polarizer

**DOI:** 10.21203/rs.3.rs-8474834/v1

**Published:** 2026-01-06

**Authors:** Arash Karimi, Zachery B. Harris, M. Hassan Arbab

**Affiliations:** Department of Biomedical Engineering, Stony Brook University, NY, USA.

**Keywords:** Terahertz polarimetry, polarizer, leaky polarizer, calibration

## Abstract

Polarization-sensitive measurements provide rich information about material properties and enable a wide range of applications. Accurate calibration of polarimetry and ellipsometry systems is especially important in broadband spectral imaging instruments, where the performance of polarizers varies with frequency and manufacturing tolerances can introduce systematic errors. Terahertz time-domain polarimetry (THz-TDP) offers amplitude and phase information across a broad spectral range that encompasses many low-energy resonances of chemicals, yet THz polarimetry remains less developed than its infrared and optical counterparts. In this work, we present a generalized *in situ* calibration technique for THz-TDP imaging systems that uses a rotating polarizer placed over a reference mirror. The method accounts for the leaky, frequency-dependent response of the wire-grid polarizer and simultaneously extracts the polarizer response and system calibration parameters from the same measurement. We implement the approach on a handheld polarimetric THz scanner and demonstrate that including a leaky polarizer model yields more consistent and accurate calibration parameters across spectrum and the field of view, as compared to a simple model of an ideal polarizer. The method is validated using two wire-grid polarizers with different extinction ratios, illustrating that an accurate *in situ* calibration can be achieved even when the polarizer response is imperfect and unknown a priori.

## I. INTRODUCTION

Polarization-sensitive techniques, including polarimetry and ellipsometry, are powerful methods for characterizing material properties. By detection of changes in the state of polarization upon reflection, transmission, or scattering, these techniques provide access to subtle properties that are often invisible to purely intensity based optical measurements^1–4^. Conventional polarimetry and ellipsometry systems have been widely used in the IR to UV parts of the spectrum to study thin films^5–7^, dielectric materials^8^, anisotropic media^9^, biomedical samples^10^, metamaterials^11^ and other imaging applications^12^. In recent years, the extension of polarization-sensitive techniques into the terahertz (THz) frequency range (i.e., between 0.1 and 10 THz) has attracted substantial interest^13–16^. The sensitivity of THz radiation to structural chirality enables probing dichroism signatures associated with biomolecules^17,18^, metamaterials^19,20^, and chiral lattice vibrations^21^. Measurements of circular birefringence and optical activity facilitate the characterization of anisotropic crystals^22,23^, polymers^24,25^, and composite materials^26,27^. Polarimetric measurement of electromagnetic scattering further extends these capabilities to quantification of surface roughness through statistical optics^28^ and structural inhomogeneity in biological tissue^29,30^ and skin appendages^31^. Additionally, advanced THz Mueller matrix ellipsometers have enabled accurate characterization of various material systems^32–34^.

The polarization state of the terahertz light can be readily reconstructed by the field strength of two orthogonal components of the light measured using the terahertz time-domain spectroscopy (THz-TDS) technique, which simultaneously provides both amplitude and phase information^35–37^. The polarization sensitivity of photoconductive antennas, arising from the dependence of THz detection on the relative orientation between light polarization and the electrode gap, has been combined with external polarizers to separate the orthogonal components of the THz field^38–40^. The accuracy of these systems is thus limited by the loss or leaky behavior of the external polarizers or the cross talk between the two THz detection channels. Therefore, although terahertz time-domain polarimetry (THz-TDP) provides rich information about the material properties, accurate measurements rely on careful calibration. Full polarimetric characterization of each optical element may not always be feasible, and a small misalignment can cause significant miscalibration of the system. Therefore, it is often ideal to calibrate a THz-TDP system using a well characterized *in situ* sample^41,42^.

Previously, we introduced the polarimetric version of our THz Portable HAndheld Spectral Reflection (PHASR) Scanner that records the orthogonal polarization components of the reflected THz pulse using two detectors separated by a wire grid polarizer beam splitter^43^. We also introduced an *in situ* calibration model that utilizes a rotating polarizer in front of a mirror^44^. We showed that using this approach both the incident polarization state of the THz light on the sample in the reflection geometry, as well as the Jones matrix of the scanner, can be quantified in both spectral and spatial dimensions. However, to simplify the model, we assumed that the polarizer is ideal, which means that it has no optical loss or polarization leakage. However, commercial THz polarizers can be imperfect and have frequency-dependent transmission spectra in both orthogonal polarizations of the light, which depend on their structure and operating wavelength. Liquid-crystal polarizers (LCPs) and wire grid polarizers (WGPs) are widely used in the THz regime. LCPs offer a high extinction ratio, but their loss increases with frequency^45^. In contrast, free-standing WGPs exhibit low loss due to the high conductivity of the metallic wires, but their extinction ratio decreases with decreasing operating wavelength^46,47^. Due to the dependence of the frequency response of a polarizer, the finite extinction ratio and the loss of these optical elements play a crucial role in broadband THz systems^47^.

In this work, the imperfect response of a WGP is incorporated into the calibration of the polarimetric THz PHASR Scanner to improve the accuracy and feasibility of the technique over a wide bandwidth. Additionally, an *in situ* calibration technique based on ADAM gradient descent on a Riemann complex surface is proposed, which extracts the frequency response of the polarizer along with the calibration of the polarimetric system simultaneously. In other words, using this new model, an accurate calibration of a THz polarimetry system can be achieved even when the spectral and spatial performance of the imperfect polarizer is unknown. The importance of having an accurate model of the polarizer is shown by comparing the calibration parameters using a simple ideal model with no loss and no leakage, and a more realistic model of the polarizer that accounts for its leakage. Furthermore, the independence of the calibration parameters from the imperfect frequency response of the polarizer used for calibration is verified by using two WGPs of different extinction ratios.

## II. MATERIALS AND METHODS

### A. Imperfect Frequency Response of Polarizers

Polarizers are widely used in polarimetry and ellipsometry to separate one linear polarization component of the light from its orthogonal counterpart. An ideal WGP can be described as an optical element that transmits the electric field component perpendicular to the metal wires and completely reflects the parallel polarization to the wires. The simple Jones matrix of an ideal polarizer in the wire basis can be written as

(1) $${\text{J}}_{\text{P}}^{\left(s\right)}=\left[\begin{array}{ll} 1 & 0\\ 0 & 0 \end{array}\right].$$

In practice, however, the attenuation and transmission of the polarization parallel to the wires and the loss of the polarization perpendicular to the wires are non-zero and depend on the geometry of the polarizer and the operating frequency. A more realistic version of the Jones matrix in Eq. (1) is therefore,

(2) $${\text{J}}_{\text{P}}^{\left(l\right)}=\left[\begin{array}{cc} t_{⊥} & 0\\ 0 & t_{\|} \end{array}\right],$$

where $t_{⊥}$ and $t_{\|}$ are the complex transmission coefficients of the electric field components perpendicular and parallel to the wires, respectively. The superscripts (s) and (l) will be used throughout this paper to refer to the simple and leaky models of a polarizer given in Eq. (1) and Eq. (2), respectively. The coefficients $t_{⊥}$ and $t_{\|}$ are functions of the wire period p, the wire diameter d, and the operating free-space wavelength λ. The ideal case with $t_{⊥}=1$ and $t_{⊥}/t_{\|}\to \infty$ can be approximated only over a limited frequency band by an appropriate choice of d and p. For broadband systems, ${\text{J}}_{\text{P}}$ is far from ideal as λ approaches p.

**FIG. 1.**
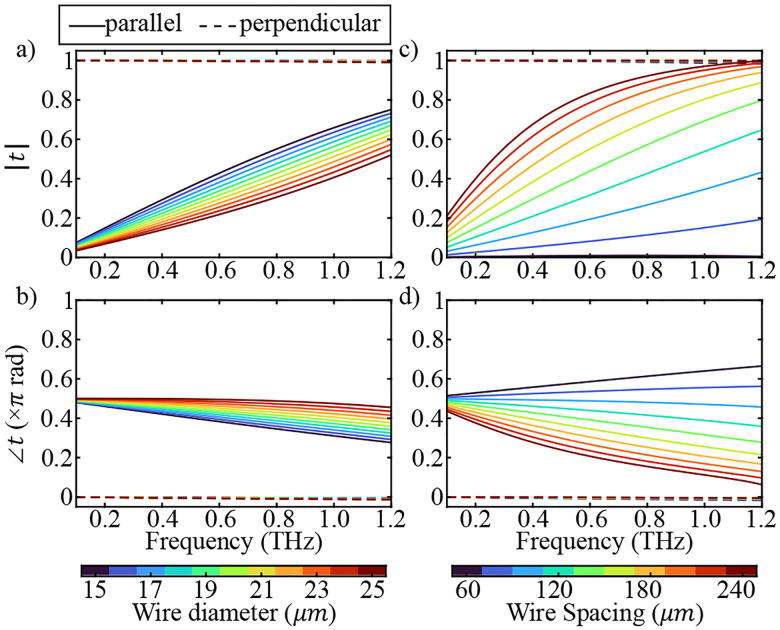
(a) Amplitude and (b) phase frequency response of a WGP to the electric field polarization parallel to the wires $t_{\|}$ (solid lines) and perpendicular to the wires (dashed lines) with constant wire period p=100μm and varying wire diameter between 15–25μm. (c) Amplitude and (d) phase frequency response dependence of $t_{\|}$ and $t_{⊥}$ to p varying between 60–240μm with a constant d=20μm.

As a practical approximation, an impedance analogy can be used to derive analytical expressions for $t_{⊥}$ and $t_{\|}$ of a free-standing WGP, as described elsewhere^47^. As an example, Fig. 1 shows the frequency response of $t_{\|}$ and $t_{⊥}$ at various d and p using the impedance model. Figs. 1(a) and (b) show the amplitude and phase frequency response of $t_{\|}$ (solid lines) and $t_{⊥}$ (dashed lines) of a WGP with p=100μm and varying d between 15–25μm. Figs. 1(c) and (d) show the dependence of the amplitude and phase of $t_{\|}$ and $t_{⊥}$ on p with a constant d=20μm. As can be seen, $t_{⊥}$ is nearly constant and almost equal to 1 with a phase shift of 0 due to the high conductivity of metal wires. However, the amplitude and phase of $t_{\|}$ drastically vary with d and p throughout the spectrum. Therefore, Eq. (2) can be modified to

(3) $${\text{J}}_{\text{P}}^{\left(l\right)}=\left[\begin{array}{cc} 1 & 0\\ 0 & t_{\|}/t_{⊥} \end{array}\right]=\left[\begin{array}{cc} 1 & 0\\ 0 & \eta e^{j\delta } \end{array}\right],$$

where η and δ are the leakage (reciprocal of the extinction ratio) and the phase retardance of the electric field parallel to the orientation of the wires^48^.

**FIG. 2.**
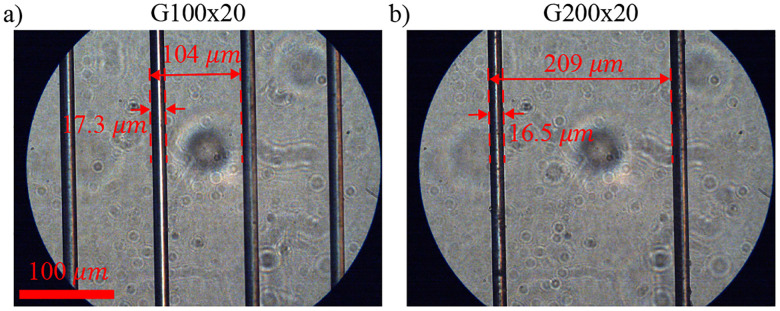
Microscopic images of (a) G100x20 and (b) G200x20 WGPs from a random region with measured wire diameter and wire period.

In this study, two tungsten WGPs with a nominal wire diameter of 20μm and a wire period of 100μm and 200μm, namely G100x20 and G200x20, respectively, are used (Microtech Instrument inc. free-standing wire grid polarizer). The wire period and wire diameter of the two WGPs are measured from 10 random regions using an optical microscope. The G200x20 WGP had a wire diameter of 18.5±1.2μm and a wire period of 205±5μm, and the G100x20 WGP had a wire diameter of 17.3±0.7μm and a wire period of 101.4±2.6μm. Examples of microscopic images of these two WGPs are shown in Fig. 2.

To verify the impedance model, transmission measurements of the two WGPs were performed with a pair of fiber-coupled photoconductive antennas (PCAs) operating with a delay stage (TeraSmart, Menlo Systems Inc., Newton, NJ, USA). $t_{\|}$ can be measured by dividing the transmitted spectrum from the WGP with the wires being parallel to the incident polarization over the spectrum of a reference pulse. In this transmission measurement, because PCAs produce quasilinearly polarized light^38^, a second G100x20 WGP aligned to pass the main polarization of the PCA emitter was included in the incident beam path to minimize the effect of cross-talk between the two polarizations^49^.

### B. Polarimetric THz PHASR Scanner

**FIG. 3.**
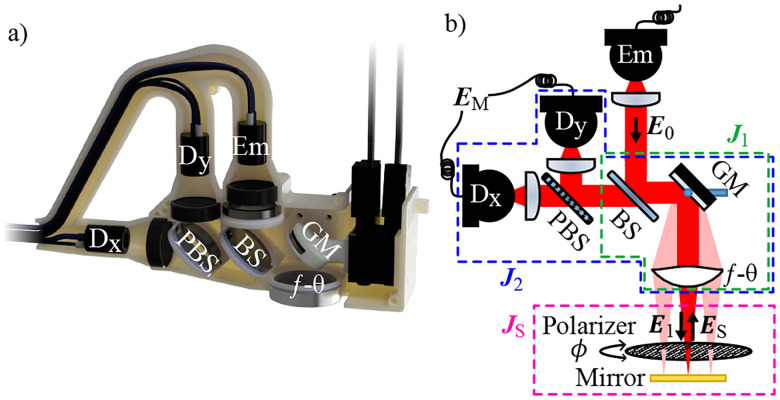
(a) Photograpth and (b) schematic of the polarimetric THz PHASR Scanner with a WGP in front of a mirror in the sample place for calibration purposes.

The polarimetric THz scanner consists of a PCA emitter rotated to a 45° angle, and a pair of PCA detectors oriented to two orthogonal polarizations. The TDS system operates using the Electronically Controlled OPtical Sampling (ECOPS) technique^50^ (TeraFlash smart, Toptica Photonics AG, Germany). Fig. 3 shows the schematic and 3D rendering of the PHASR Scanner and its internal components. The collimated beam ${\text{E}}_{0}$ from the emitter is reflected from the beam splitter and is steered across the scanning lens via a motorized gimbal mirror (GM). The collimated beam is then focused on the sample using a custom f-θ lens^51^. The s- and p-polarizations of the beam at the reflection from the GM change with the mirror’s orientation. With the gimbal geometry used here, both components are affected by rotation of the GM about its azimuthal axis. However, elevation rotation of the GM affects only the p-polarization component of the light. As a result, the polarization of the light passing through the f-θ lens changes as the beam is steered and, therefore, the polarization incident on the sample varies across the field of view (FOV)^44,52^. As shown in Fig. 3(b), The incident electric field on the sample can be expressed as ${\text{E}}_{1}={\text{J}}_{1}{\text{E}}_{0}$, where ${\text{J}}_{1}$ is the Jones matrix of the system from the emitter to the sample. The reflected beam from the sample passes through the same path and is further separated into two orthogonal polarizations by a polarizing beam splitter (PBS) to be recorded by the two detectors, D_x_ and D_y_. The measured electric field ${\text{E}}_{\text{M}}$ is given by

(4) $$\left[\begin{array}{l} E_{\text{M},x}\\ E_{\text{M},y} \end{array}\right]={\text{J}}_{2}{\text{J}}_{\text{S}}{\text{E}}_{1}=\left[\begin{array}{ll} s_{xx} & s_{xy}\\ s_{yx} & s_{yy} \end{array}\right]{\text{J}}_{\text{S}}\left[\begin{array}{l} E_{1,x}\\ E_{1,y} \end{array}\right],$$

where as defined in Fig. 3b ${\text{J}}_{2}$ is the Jones matrix of the scanner on the reflection path from the sample to the detectors, and ${\text{J}}_{\text{S}}$ is the Jones matrix of the sample. Due to the multiplicative non-uniqueness of ${\text{E}}_{\text{M}}={\text{J}}_{2}{\text{J}}_{\text{S}}{\text{E}}_{1}$, the $s_{xx}$ element in ${\text{J}}_{2}$ is factorized to reduce the number of unknown parameters of the system to five. In other words, we can modify ${\text{J}}_{2}$ and ${\text{E}}_{1}$ to ${\text{J}}_{2}^{\prime }$ and ${\text{E}}_{1}^{\prime }$ using,

(5a) $$\begin{array}{c} {\text{J}}_{2}^{\prime }=\frac{{\text{J}}_{2}}{s_{xx}}=\left[\begin{array}{cc} 1 & s_{1}\\ s_{2} & s_{3} \end{array}\right], \end{array}$$

(5b) $${\text{E}}_{1}^{\prime }=s_{xx}{\text{E}}_{1}=\left[\begin{array}{c} E_{1,x}^{\prime }\\ E_{1,y}^{\prime } \end{array}\right],$$

Therefore, Eq. (4) can be replaced by

(6) $$\left[\begin{array}{l} E_{\text{M},x}\\ E_{\text{M},y} \end{array}\right]={\text{J}}_{2}^{\prime }{\text{J}}_{\text{S}}{\text{E}}_{1}^{\prime }=\left[\begin{array}{cc} 1 & s_{1}\\ s_{2} & s_{3} \end{array}\right]{\text{J}}_{\text{S}}\left[\begin{array}{l} E_{1,x}^{\prime }\\ E_{1,y}^{\prime } \end{array}\right].$$

### C. Calibration of the Polarimetric THz PHASR Scanner

We have previously shown that calibration of this system is performed by placing a rotating WGP in front of a mirror in the sample position^44^. The expanded Jones matrix of this sample is^53,54^

(7a) $${\text{J}}_{\text{S}}(\phi )=\text{R}(-\phi ){\text{J}}_{\text{p}}{\text{J}}_{\text{Mir.}}{\text{J}}_{\text{p}}\text{R}(\phi ),$$

(7b) $$\text{R}\left(\phi \right)=\left[\begin{array}{cc} \text{cos}\left(\phi \right) & \text{sin}\left(\phi \right)\\ -\text{sin}\left(\phi \right) & \text{cos}\left(\phi \right) \end{array}\right],$$

(7c) $${\text{J}}_{\text{Mir.}}=\left[\begin{array}{cc} -1 & 0\\ 0 & -1 \end{array}\right],$$

where ${\text{J}}_{\text{P}}$ is the Jones matrix of the WGP, as explained in Section II A. The rotation angle of the WGP ϕ is chosen to increase with counterclockwise rotation, starting at ϕ=0 to have the wires along with the vertical polarization. By substituting Eq. (7a) into Eq. (6), and further decomposing the angle-dependent parameters from ${\text{J}}_{2}^{\prime }$ and ${\text{E}}_{1}^{\prime }$, a matrix equation system is obtained, given by,

(8) $$\left[\begin{array}{c} E_{\text{M},x}\\ E_{\text{M},y} \end{array}\right]=-\left[\begin{array}{cccccc} {\text{cos}}^{2}\left(\phi \right) & \text{cos}\left(\phi \right)\text{sin}\left(\phi \right) & {\text{sin}}^{2}\left(\phi \right) & 0 & 0 & 0\\ 0 & 0 & 0 & {\text{cos}}^{2}\left(\phi \right) & \text{cos}\left(\phi \right)\text{sin}\left(\phi \right) & {\text{sin}}^{2}\left(\phi \right) \end{array}\right]\left[\begin{array}{c} A_{1}\\ A_{2}\\ A_{3}\\ A_{4}\\ A_{5}\\ A_{6} \end{array}\right],$$

where vector A is the auxiliary vector that consists of the calibration parameters. Eq. (8) is under determined and requires at least three sets of independent measurements to calculate A. In this experiment, we acquired measurements with ϕ between 0° and 170° with a step size of 10°. Depending on the choice of the model (simple or leaky) for the WGP, the auxiliary vector can be composed of two different sets of calibration parameters, as given by,

(9a) $$\left[\begin{array}{c} A_{1}\\ A_{2}\\ A_{3}\\ A_{4}\\ A_{5}\\ A_{6} \end{array}\right]=\left[\begin{array}{c} E_{1,x}^{\prime (s)}\\ E_{1,y}^{\prime (s)}+s_{1}^{\left(s\right)}E_{1,x}^{\prime (s)}\\ s_{1}^{\left(s\right)}E_{1,y}^{\prime (s)}\\ s_{2}^{\left(s\right)}E_{1,x}^{\prime (s)}\\ s_{2}^{\left(s\right)}E_{1,y}^{\prime (s)}+s_{3}^{\left(s\right)}E_{1,x}^{\prime (s)}\\ s_{3}^{\left(s\right)}E_{1,y}^{\prime (s)} \end{array}\right],$$

and

(9b) $$\left[\begin{array}{c} A_{1}\\ A_{2}\\ A_{3}\\ A_{4}\\ A_{5}\\ A_{6} \end{array}\right]=\left[\begin{array}{c} E_{1,x}^{\prime (l)}+\zeta s_{1}^{\left(l\right)}E_{1,y}^{\prime (l)}\\ \left(E_{1,y}^{\prime (l)}+s_{1}^{\left(l\right)}E_{1,x}^{\prime (l)}\right)(1-\zeta )\\ \zeta E_{1,x}^{\prime (l)}+s_{1}^{\left(l\right)}E_{1,y}^{\prime (l)}\\ s_{2}^{\left(l\right)}E_{1,x}^{\prime (l)}+\zeta s_{3}^{\left(l\right)}E_{1,y}^{\prime (l)}\\ \left(s_{2}^{\left(l\right)}E_{1,y}^{\prime (l)}+s_{3}^{\left(l\right)}E_{1,x}^{\prime (l)}\right)(1-\zeta )\\ \zeta s_{2}^{\left(l\right)}E_{1,x}^{\prime (l)}+s_{3}^{\left(l\right)}E_{1,y}^{\prime (l)} \end{array}\right].$$

For simplicity, $\zeta =\eta^{2}e^{j2\delta }$ is used in these equations. Note that Eq. (9a) and Eq. (9b) are the same if ζ=0. The general analytical solution to both Eq. (9a) and Eq. (9b) can be derived after algebraic manipulations, which is given by,

(10a) $$E_{1,x}^{\prime }=\frac{A_{1}-\zeta A_{3}}{1-\zeta^{2}},$$

(10b) $$E_{1,y}^{\prime }=\frac{2\left(A_{1}-\zeta A_{3}\right)\left(A_{3}-\zeta A_{1}\right)}{\left(A_{2}(1+\zeta )+\sqrt{\Delta }\right)\left(1-\zeta^{2}\right)},$$

(10c) $$s_{1}=\frac{A_{2}(1+\zeta )+\sqrt{\Delta }}{2\left(A_{1}-\zeta A_{3}\right)},$$

(10d) $$s_{2}=\frac{A_{4}-\zeta A_{6}}{A_{1}-\zeta A_{3}},$$

(10e) $$s_{3}=\frac{\left(A_{6}-\zeta A_{4}\right)\left(A_{2}(1+\zeta )+\sqrt{\Delta }\right)}{2\left(A_{1}-\zeta A_{3}\right)\left(A_{3}-\zeta A_{1}\right)},$$

where ${\text{E}}_{1}^{\prime (s)}$ and ${\text{J}}_{2}^{\prime (s)}$ can be calculated simply by setting ζ=0. The parameter Δ is a discriminant of a quadratic equation that arises during the solution of the auxiliary vector to the calibration parameters, and is defined as

(11) $$\Delta =A_{2}^{2}(1+\zeta {)}^{2}-4\left(A_{1}-\zeta A_{3}\right)\left(A_{3}-\zeta A_{1}\right).$$

The presence of a complex square root term in $E_{1,y}^{\prime },s_{1}$, and $s_{3}$ defines two possible solutions due to the inherent branch ambiguity of the square root.

In case of a known ζ, Eq. (9b) has 5 unknown parameters and 6 equations, 5 of which are used in deriving Eqs. (10). Thus, the unused equation (in this case, $A_{5}$) can be used to choose the correct root of the quadratic equation. In other words, the calculated calibration parameters from Eqs. (10) can be substituted on the right-hand side of Eq. (9b) to form two calculated values of $A_{5}$ for each root. The solution that minimizes the difference between the calculated $A_{5}$ (right side) and the measured $A_{5}$ (left side) would be the correct answer. Although this allows one to simply assign the value of ζ in Eqs. (10) to obtain the calibration parameters, it requires a separate measurement of the calibrating WGP. However, in many applications, an *in situ* measurement is preferred due to the ease of workflow and real-time analysis^55,56^. If ζ is treated instead as an unknown parameter, Eq. (9b) has 6 equations and 6 unknown values. Therefore, Eq. (10) can be substituted into the fifth row of Eq. (9b) to form the calculated $A_{5}$, which is given as

(12) $${\overset{ˆ}{A}}_{5}=\frac{2\left(A_{3}-\zeta A_{1}\right)\left(A_{4}-\zeta A_{6}\right)}{A_{2}(1+\zeta {)}^{2}+\sqrt{\Delta }(1+\zeta )}+\frac{\left(A_{2}(1+\zeta )+\sqrt{\Delta }\right)\left(A_{6}-\zeta A_{4}\right)}{2\left(A_{3}-\zeta A_{1}\right)(1+\zeta )}.$$

By minimizing the error between $A_{5}$ and ${\overset{ˆ}{A}}_{5},\zeta$ can be extracted from Eq. (12). Therefore, the objective of this problem is given by

(13a) $$\underset{\zeta \in C}{\text{min}\;}C\left(\text{A},\zeta \right),$$

(13b) $$C\left(\text{A},\zeta \right)=\sum_{x}\sum_{y}\Psi \Psi^{*},$$

(13c) $$\Psi \left(\text{A},\zeta \right)={\overset{ˆ}{A}}_{5}-A_{5}.$$

**FIG. 4.**
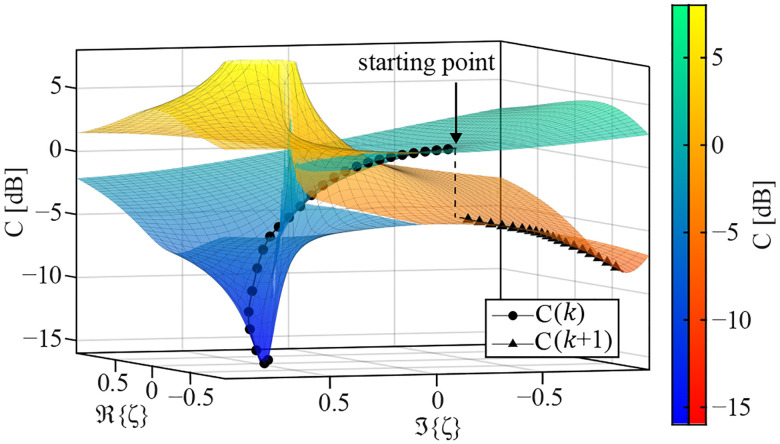
Cost function minimization on a two-sheet Riemann surface of C. The two color maps indicate the odd and even unwrapping factor k.

By identifying ζ=α+jβ where $(\alpha ,\beta )\in R^{2}$, the 1D complex minimization problem can be converted into a 2D real-valued Riemann surface^57^. In addition, although A is a function of frequency and space, ζ is only a function of frequency. Thus, the space dependence of the cost function can be removed by performing a summation of the values of $\Psi \Psi^{*}$ in space. This summation in space also suppresses the measurement noise. The Adaptive Moment Estimation (ADAM) optimization gradient descent^58^ was used to minimize the cost function in Eq. (13a). The utilization of the ADAM optimizer has several benefits in solving this problem. Firstly, the values of C(A,ζ) vary by several orders of magnitude along the frequency axis. By normalizing the gradient, the ADAM optimizer removes the bias of C(A,ζ) in frequency. In addition, the momentum-based gradient is less susceptible to noise and non-smooth cost functions. The key consideration for solving Eq. (13a) is tracking the correct Riemann sheet over the iterations of minimization. Since $\sqrt{\Delta }$ is the origin of the ambiguity in C, it is best to track the continuous path on the Riemann surface of $\sqrt{\Delta }$. The polar form of $\sqrt{\Delta }$ is given as

(14) $$\sqrt{\Delta }=\sqrt{\left|\Delta \right|}e^{j\frac{∠\Delta }{2}+k\pi },$$

where k∈Z is the unwrapping factor and depending on whether it is odd or even, $\sqrt{\Delta }$ can have a different sign. This continuity tracking is based on minimizing the Euclidean distance of the previous $\sqrt{\left|\Delta_{i-1}\right|}e^{j\frac{∠\Delta_{i-1}}{2}+k\pi }$ with the two possible next points $\sqrt{\left|\Delta_{i}\right|}e^{j\frac{∠\Delta_{i}}{2}+k\pi }$ and $\sqrt{\left|\Delta_{i}\right|}e^{j\frac{∠\Delta_{i}}{2}+(k+1)\pi }$. With each detection of discontinuity, k is increased by one. Since $\sqrt{\Delta }$ is a function of α,β, frequency, and space, this continuity tracking should be performed along each dimension. At each iteration with a constant α and β, each pixel provides a spectrum that can easily be unwrapped. The unwrapping operation in space is more complicated, and a flood-fill algorithm is utilized, which starts unwrapping the pixels from the center of the FOV, where the SNR is highest^59^. Lastly, at each iteration with new α and β, the continuity of $\sqrt{\Delta }$ should be ensured by comparing it with the previous point. Note that the two-sheet Riemann surface is only created in the (α,β) dimension, and in the frequency and space create two separate roots. Due to the inherent phase ambiguity of the problem, two starting points with different values of k (different signs) are chosen to ensure convergence. Fig. (4) shows an example of the optimization on Riemann surface at 0.5 THz. As can be seen, at least one of the starting points with different starting values of k converges to the global minima of the cost function.

## III. RESULTS

### A. Characterization of the Polarizers

**FIG. 5.**
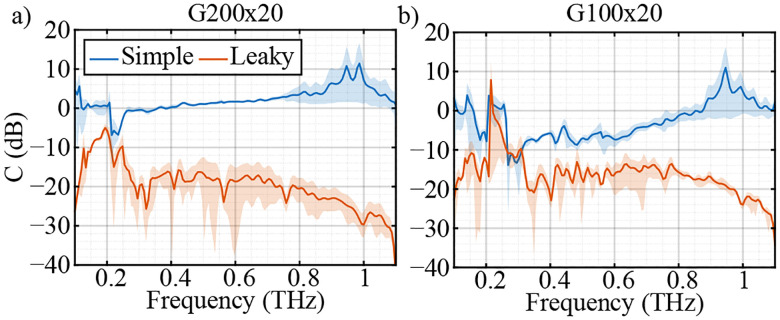
frequency-dependent cost function C calculated from the error between $A_{5}$ and ${\overset{ˆ}{A}}_{5}$, with ζ=0 (blue curve) and the optimized ζ value (red curve) using the calibration set with (a) G200x20 and (b) G100x20 WGPs. The shaded area represents the range of error within a 10 mm×10 mm area in the FOV.

Fig. 5 shows the cost functions of the two calibration sets, measured with G200x20 and G100x20 polarizers. The cost functions are calculated using both the simple model of the WGPs, i.e., with ζ=0 (blue curve), and the extracted ζ value (red curve). It is evident that the use of the optimization method significantly reduces the error between $A_{5}$ and ${\overset{ˆ}{A}}_{5}$ by about 20 dB for the G200x20 WGP calibration set, and approximately 10 dB for that of the G100x20 polarizer. The exact amount of error reduction depends on the SNR of the measurements, stopping criteria and the step size in ADAM optimization, as well as the inherent leakage of the polarizer itself.

**FIG. 6.**
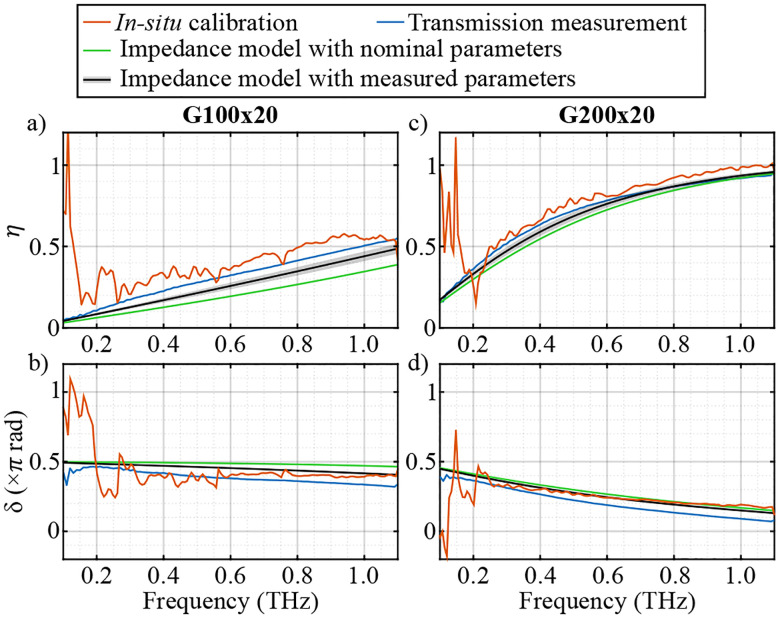
(a) The leakge η and (b) phase retardance δ of the G100x20, and (c) η and (d) δ of the G200x20 WGP extracted from the *in situ* calibration measurement with gradient descent (red), transmission measurement of the WGPs (blue), the impedance model with nominal wire diameter d and wire period p (green) and impedance model with measured d and p (black).

Fig. 6 shows η and δ for the two calibration sets using G100x20 and G200x20. The red curves are the values extracted from the *in situ* calibration using the optimization method. In addition, as discussed in Section. II A, η and δ of the WGPs can be attained by a simple transmission measurement,

(15) $$\eta e^{j\delta }=\frac{E_{\|}}{E_{⊥}},$$

where $E_{\|}$ and $E_{⊥}$ are the complex spectra of the recorded electric field with the polarization parallel and perpendicular to the wires of the WGP, respectively. η and δ of the two polarizers measured in the transmission setup are shown as blue curves in Fig. 6. In addition, these parameters are calculated from the impedance model using the nominal (green) and measured (black) values of the wire diameter d and the wire period p. As can be seen, there is excellent agreement between the parameters extracted from various methods within the bandwidth of the system. In particular, as η→1 and δ→0 the denominators in Eq. (10a) and Eq. (10b) approach zero, and the calibration problem becomes ill-conditioned. In other words, in this case the WGP ceases to provide meaningful rotation-angle-dependent contrast for calibration purposes.

In transmission measurements, the ratio of $E_{⊥}$ for both G200x20 and G100x20 to a reference electric field in the absence of a sample WGP was measured to be nearly 1. The negligible loss of the polarizers is due to the high conductivity of the wires parallel to the polarization of light.

### B. Calibration Results

In the following, four scenarios for extracting the calibration parameters ${\text{E}}_{1}^{\prime }$ and ${\text{J}}_{2}^{\prime }$ are compared. For each of the two WGPs, G200x20 and G100x20, Eq. (10) is solved using the simple model where ζ=0 and using the leaky model where ζ is calculated from the optimization method described in Section II C.

The two complex values of the incident electric field ${\text{E}}_{1}^{\prime }$ Jones vector can also be expressed as four components of the Stokes vector, defined as

(16a) $$I={\left|E_{1,x}^{\prime }\right|}^{2}+{\left|E_{1,y}^{\prime }\right|}^{2},$$

(16b) $$Q={\left|E_{1,x}^{\prime }\right|}^{2}-{\left|E_{1,y}^{\prime }\right|}^{2},$$

(16c) $$U=2R\left(E_{1,x}^{\prime }E_{1,y}^{\prime *}\right),$$

(16d) $$V=-2J\left(E_{1,x}^{\prime }E_{1,y}^{\prime *}\right),$$

where I,Q,U, and V are the total intensity of the electric field, the difference in the intensity of vertical/horizontal, ±45°, and right/left-handed polarizations, respectively. Q,U and V are normalized by the intensity spectrum I, as is a common practice in polarimetry and ellipsometry. Note that although ${\text{E}}_{1}^{\prime }$ is normalized by $s_{xx}$ in Eq. (5b), only I has a factor of ${\left|s_{xx}\right|}^{2}$, while the other three Stokes parameters Q/I,U/I and V/I, are independent of this factorization.

**FIG. 7.**
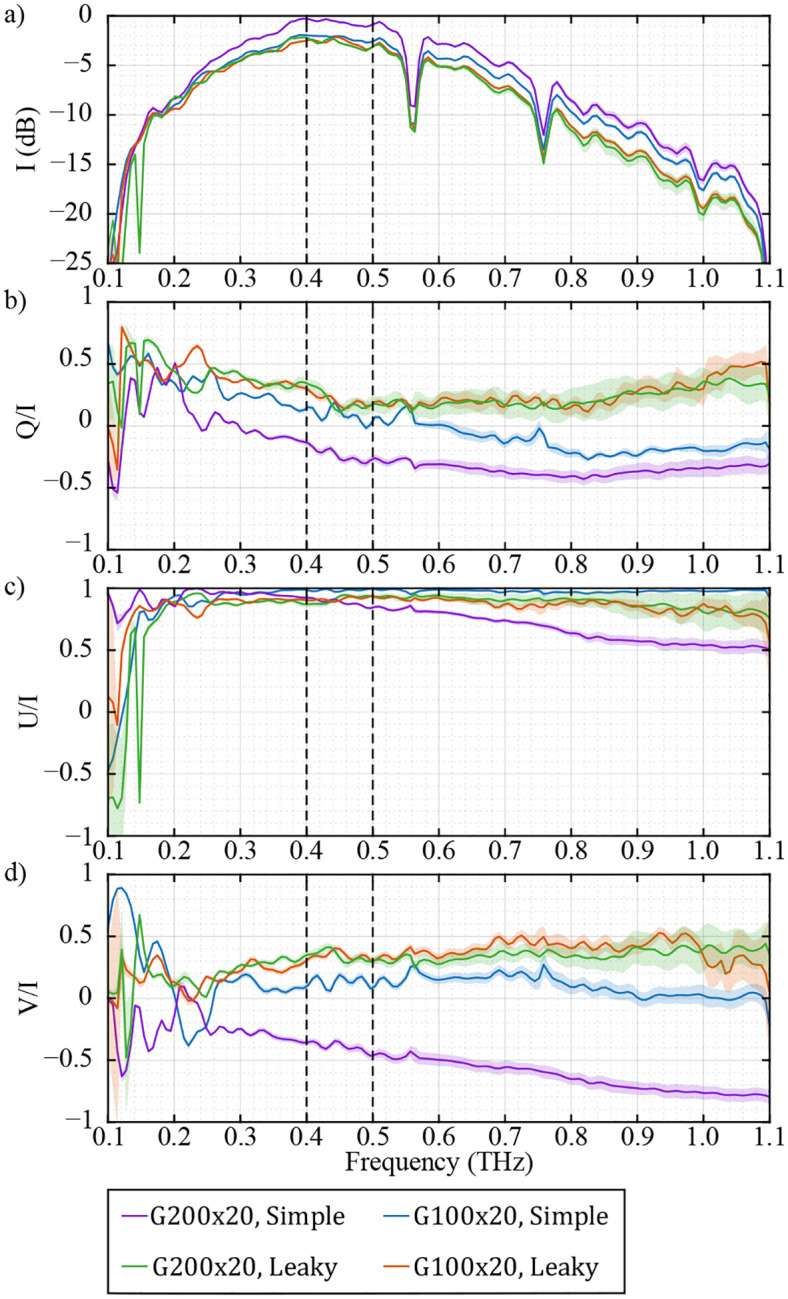
Frequency-dependent normalized Stokes vector (a) I, (b) Q/I, (c) U/I, and (d) V/I of the incident electric field ${\text{E}}_{1}^{\prime }$. The solid lines and shaded area represents the mean and standard deviation of the values from a 9-mm vertical line in the center of the FOV. The purple and blue curves are extracted from the simple model of the two WGPs G200x20 and G100x20, respectively, with the assumption of no leakage. The green and red curves are the extracted values with the leaky model of the two WGPs G200x20 and G100x20, respectively.

Fig. 7 shows the frequency-dependent Stokes vector elements of the incident electric field ${\text{E}}_{1}^{\prime }$ for the four calibration cases. The Stokes vector elements are averaged from a 9-mm vertical line in the center of the FOV, and the shaded area represents the standard deviation of this region. In the case of using the simple model of the WGPs, the Stokes vector components of the two calibration sets with G200x20 (purple) and G100x20 (blue) polarizers have a clear discrepancy. However, the leaky model gives similar Stokes vector elements regardless of the choice of the WGP. In other words, one can anticipate that the calibration parameters of a system should not depend on the sample used to calibrate it. Using the new model, the system calibration is now independent of the choice of the leaky WGP. Note that in Fig. 7(a), the use of the leaky WGP model reduces the extracted I of the incident beam by ~2 dB at 0.4 THz, and ~3 dB at 0.8 THz. As η increases, the WGP sample leaks more cross-polarized signal to the detectors, increasing the total measured power. Thus, with the assumption of an ideal WGP (simple model), the calibrated I would be overestimated. This frequency-dependence overestimation is more clearly evident in the blue curve in Fig. 7(a). As the G100x20 WGP closely approximates the ideal model at low frequencies but not at high frequencies, we can see that the blue curve matches the red and green curves at lower frequencies (low η) and converges to the purple curve at higher frequencies (high η).

**FIG. 8.**
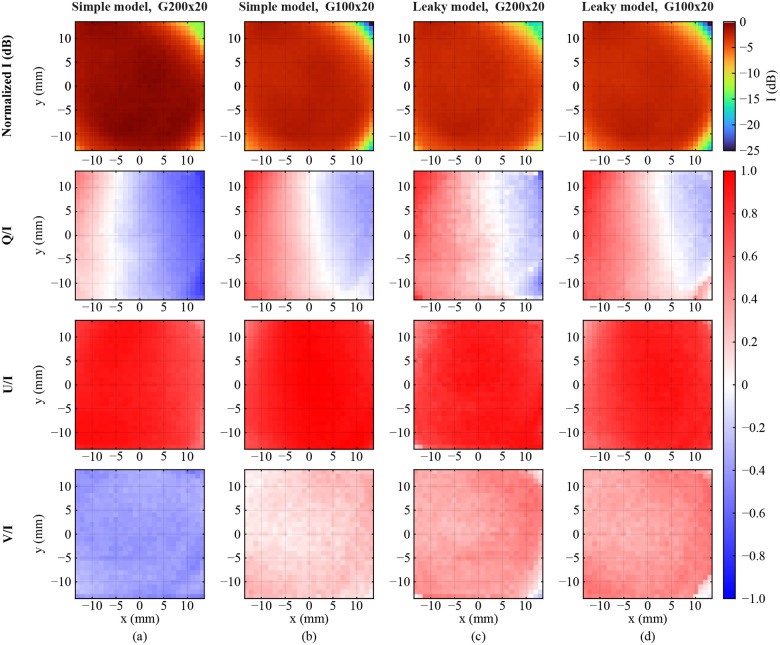
Spatial distribution of the normalized Stokes vector of the incident electric field ${\text{E}}_{1}^{\prime }$. The false color represents the average of the spectrum between 0.4–0.5 THz. The calibration was performed using the (a) G200x20 and (b) G100x20 WGPs with the assumption of simple mode for the polarizers. Calibration by (c) G200x20 and (d) G100x20 WGPs with the leaky model of the polarizers.

Figs. 8(a)–(d) show the spatial distribution of the Stokes vector parameters for cases 1–4, respectively. The false color of the figures represents the area under the curve of the spectrum of each pixel between 0.4–0.5 THz (black dashed lines in Fig. 7). As can be seen in Fig. 8(a) and Fig. 8(b), the use of the simple model of the WGPs results in a clear mismatch between the Stokes vector maps of ${\text{E}}_{1}^{\prime }$ in the two calibration sets. On the other hand, Fig. 8(c) and Fig. 8(d), which are associated with the use of the leaky WGP model in the equations, agree with each other. Note that the intensity I is nearly constant throughout the FOV, and is only reduced in the corner pixels. Q/I changes due to the movement of the GM, as discussed in Section II B. U/I has a small spatial variation and is mostly at its maximum value due to the 45° orientation of the emitter. Moreover, V/I is approximately constant throughout the FOV, and has a different sign in Fig. 8(a) compared to Figs. 8(b)–(d), meaning that neglecting the leakage resulted in wrong handedness of the incident polarization.

**FIG. 9.**
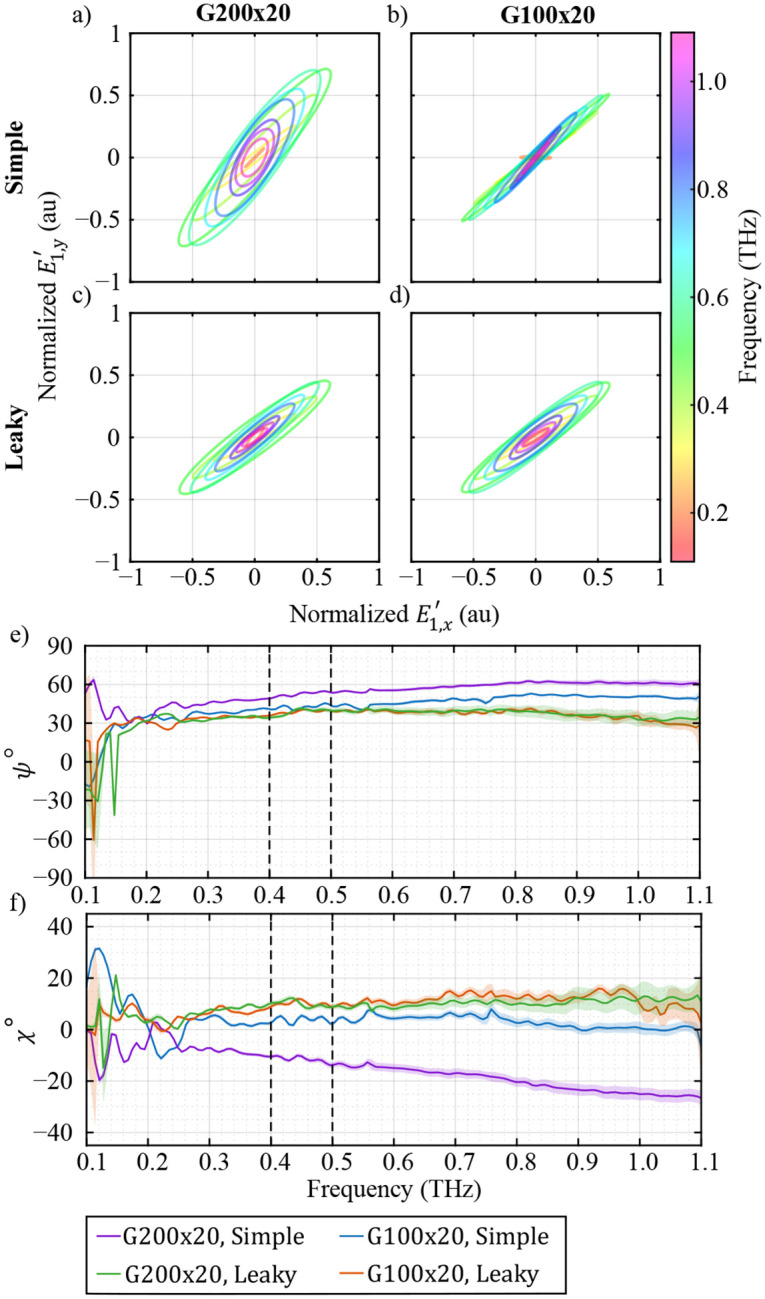
frequency-dependent polarization ellipse of ${\text{E}}_{1}^{\prime }$ of the simple model calibration of G200x20 (a) and G100x20 (b), and leaky model calibration of (c) G200x20 and (d) G100x20. frequency-dependent polarization angle (e) and ellipticity angle (f) of the 4 calibration cases. The parameters represent the average values from a 9-mm vertical line in the center of FOV.

As an alternative to the Stokes vectors, the incident electric field ${\text{E}}_{1}^{\prime }$ can be shown by the polarization ellipse representation. Figs. 9(a)–(d) show the frequency-dependent polarization ellipse of the incident electric field ${\text{E}}_{1}^{\prime }$ extracted from the simple WGP model and the leaky WGP model using G200x20 and G100x20 over a 9-mm vertical line in the center of the FOV. It can be seen that the two calibration sets using the simple model of the WGPs result in different polarization ellipses in Figs. 9(a)–(b). However, the use of the leaky model of WGPs in Figs. 9(c)–(d) extracted similar incident polarization ellipses, as expected from the schematic of the system. The polarization angle of the ellipse ψ, which is the orientation of the major axis of the polarization ellipse, can be defined as

(17) $$\psi =\frac{1}{2}{\text{tan}}^{-1}(\frac{U}{Q}),$$

and the ellipticity angle χ, which is the ratio of the minor axis of the ellipse to its major axis, is given by

(18) $$\chi =\frac{1}{2}{\text{tan}}^{-1}(\frac{V}{\sqrt{Q^{2}+U^{2}}}).$$

Note that similar to Q/I,U/I and V/I, the polarization angle ψ, the ellipticity angles χ, and the shape of the polarization ellipse do not depend on the factorization by $s_{xx}$. The frequency-dependent polarization and ellipticity angles are shown in Fig. 9(e) and Fig. 9(f), respectively. It is evident that there is a frequency-dependent overestimation in the polarization angles of the simple model of WGPs in Fig. 9(e). However, ψ of the leaky model of WGPs is relatively constant over the spectrum. Moreover, the ellipticity angles χ of the simple model of WGPs are lower than those of the leaky model in Fig. 9(f). Similar to ψ,χ shows a frequency-dependent deviation between the simple and leaky models of WGPs. The frequency-dependent deviation between the simple and leaky models of the WGPs in ψ and χ is due to the neglected leakage that increases with frequency.

**FIG. 10.**
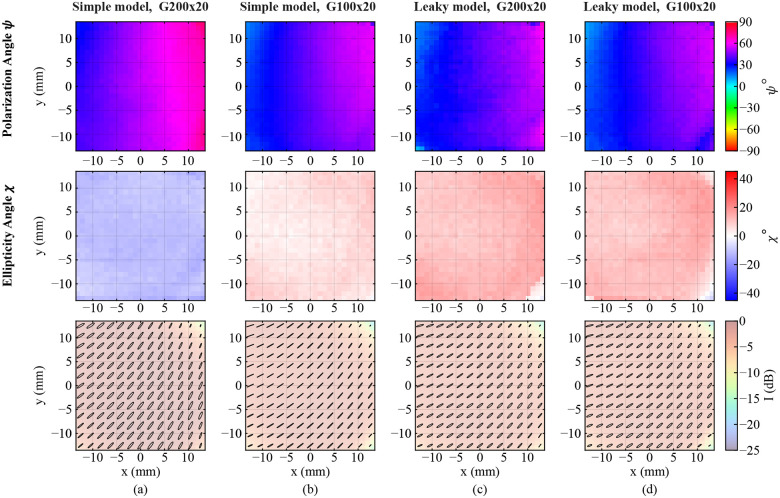
Spatial distribution of the polarization angle, ellipticity angle, and polarization ellipse of the incident electric field ${\text{E}}_{1}^{\prime }$ calibrated using (a) G200x20 simple model, (b) G100x20 simple model, (c) G200x20 leaky model, and (d) G100x20 leaky model. The false color represents the average of the spectrum between 0.4–0.5 THz.

Fig. 10 shows the spatial distribution of the polarization angle ψ, the ellipticity angle χ, and the polarization ellipses of the incident electric field (from top to bottom). In all four calibration sets, the polarization shows a large variation as the beam is swept from left to right across the FOV. This corresponds to the change in polarization due to the azimuthal rotation of the GM, discussed in Section II B. As can be seen, the two simple WGP models in Fig. 10(a) and Fig. 10(b), using the G200x20 and G100x20 WGPs, respectively, result in a clear discrepancy in the extracted polarization state of the incident beam. At y=0 in Fig. 10(a), ψ almost linearly increases from 35° on the left side of the image to 68°, while in Fig. 10(b) it increases from 24° to 55°. However, the incident polarization of the light on the sample should be independent of the polarizer used in the calibration process. This inconsistency is eliminated using the leaky model of the G200x20 and G100x20 WGPs (Figs. 10(c)–(d)). In both calibration sets with the leaky model in Figs. 10(c)–(d), ψ vary similarly from 21° on the left to 54° on the right. Note that the slope of ψ and χ along the frequency axis across the FOV is similar to that of Figs. 9(a)–(b). This is expected since the rotation of the GM is not wavelength dependent. The polarization ellipses of the incident beam over the entire FOV of the scanner are shown in the final row of Figs. 10. As expected, using the new leaky model of the two polarizers results in identical incident polarization states, which is in contrast to the simple model used for the same two polarizer.

**FIG. 11.**
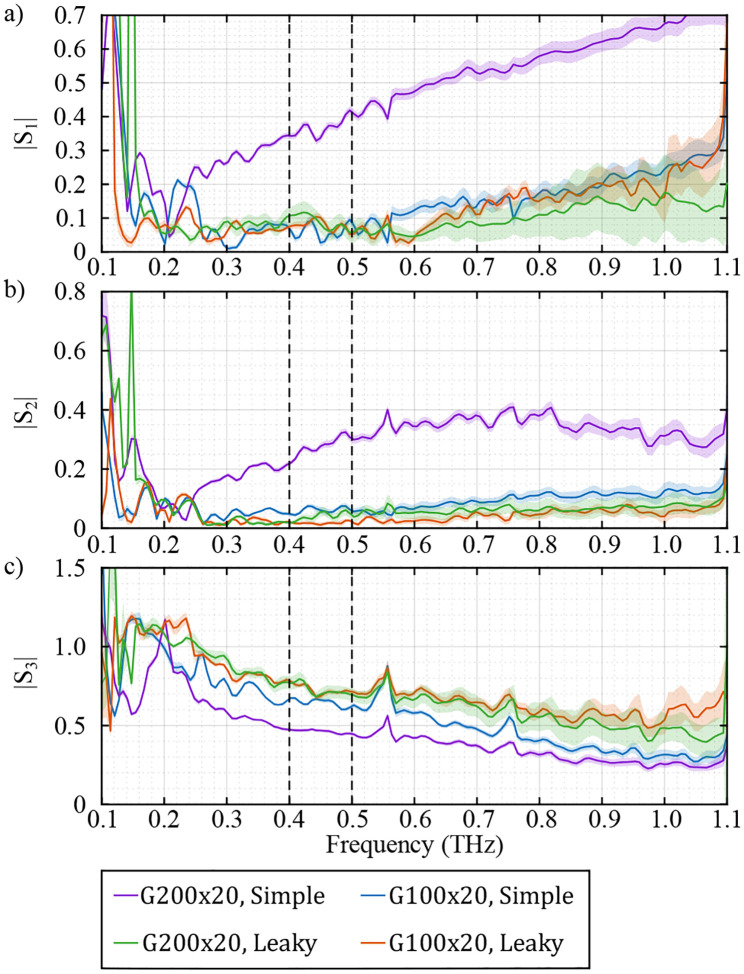
Frequency-dependent amplitude of the elements of the Jones matrix of the system in the detection path ${\text{J}}_{2}^{\prime }$. The parameters are calibrated using the simple model of G200x20 (purple), G100x20 (blue) and the leaky model of G200x20 (green) and G100x20 (red). The solid lines and shaded area indicate the mean and standard deviation of the amplitude spectra along a 9-mm vertical line in the center of the FOV.

Fig. 11 shows the frequency-dependent amplitude of the elements of ${\text{J}}_{2}^{\prime }$ averaged over a 9-mm vertical line in the center of the FOV, using the four calibration cases described above. It is evident that there is a clear mismatch between the ${\text{J}}_{2}^{\prime }$ values extracted from the two calibration sets using the simple model of the WGPs (purple and blue curves). However, using the leaky model of the WGPs, the extracted ${\text{J}}_{2}^{\prime }$ between the two calibration sets match closely with each other (red and green curves). Note that according to our earlier definitions, $s_{1}$ and $s_{2}$ capture the ratio of the cross-talk gain between the two channels, i.e., $s_{xy}$ and $s_{yx}$, to the gain of the x component $s_{xx}$. The cross-talk components are smaller than $s_{3}$, which is the ratio of the gain of y to the gain of x, i.e. $s_{yy}/s_{xx}$. It can be seen in Figs. 11(a)–(b) that neglecting the leakage of the WGPs results in increased $s_{1}$ and $s_{2}$. This is expected because the polarizer partially allows for the leakage of the cross-polarized component of the beam. Furthermore, the increasing amplitude of $s_{1}$ and $s_{2}$, and decreasing amplitude of $s_{3}$ with frequency are due to the frequency response of the PBS (defined in Fig. 3), which shows an increasing leakage of cross-polarized light with increased frequency. Additionally, the BS component (shown in Fig. 3), which contributes to ${\text{J}}_{2}^{\prime }$, is a 5-mm thick high resistive floating point silicon wafer that has a constant refractive index of n=3.416 in the bandwidth of the system, and therefore does not contribute to the frequency-dependent behavior of the components of ${\text{J}}_{2}^{\prime }$^60,61^.

**FIG. 12.**
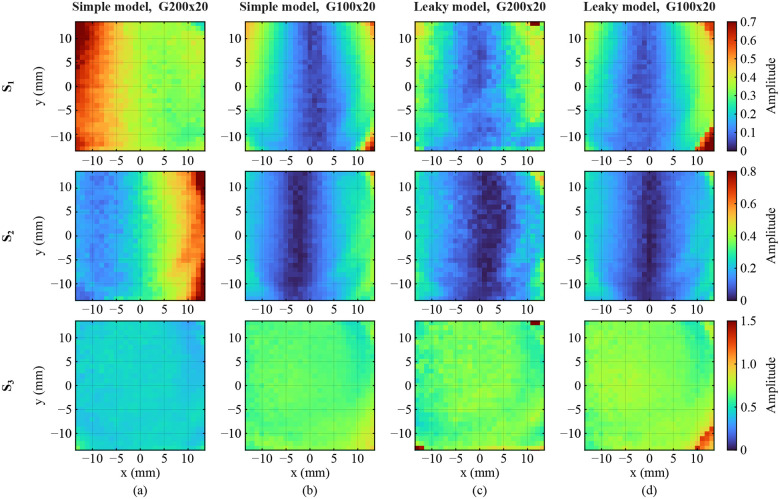
Spatial distribution of the Jones matrix of the system on detection path ${\text{J}}_{2}^{\prime }$. The parameters are calibrated using (a) the simple model of G200x20, (b) G100x20 and (c) the leaky model of G200x20 and (d) G100x20. The false color represents the average of the amplitude spectrum between 0.4–0.5 THz, as shown by dashed lines in Fig. 11.

Fig. 12 shows the spatial distribution of ${\text{J}}_{2}^{\prime }$ in the four calibration scenarios. For simplicity, the false-color shows the amplitude of the complex parameters $s_{1},s_{2}$, and $s_{3}$ averaged between 0.4–0.5 THz. As it can be seen in Figs. 12(a)–(b), the extracted ${\text{J}}_{2}^{\prime }$ of the two calibration sets using the simple WGP model do not agree with each other. In contrast, the similarity between the two sets of ${\text{J}}_{2}^{\prime }$ extracted using the leaky model, shown in Figs. 12(c)–(d) indicates that the calibration parameters are independent of the leakage of the calibrating WGPs. Note that $s_{1}$ and $s_{2}$ in Figs. 12(b)–(d) are vertically symmetric due to the azimuthal rotation of the GM, while $s_{3}$ is relatively constant in the FOV. However, due to the error of ignoring the leakage of G200x20, $s_{1}$ and $s_{2}$ in Fig. 12(a) do not show the vertically symmetric behavior.

## IV. DISCUSSION

The presented results demonstrate that incorporating a leaky model of WGPs, which accounts for frequency-dependent leakage and phase retardance of the electric field, is essential for accurate calibration of broadband polarimetry and ellipsometry systems. State-of-the-art freestanding WGPs and other types of polarizers have a variety of spectral characteristics and subsequently different frequency behavior. For narrow-band systems, the availability of a low loss and low leakage polarizer might justify the use of the simple model as described in Eq. (1). However, for broadband THz systems^62,63^, such as PCAs^64^ and air-plasma based polarimetric instruments^65^, the frequency response of the polarizer can play a crucial role in accurate calibration. The differences and similarities between the calibration parameters of the polarimetric THz PHASR Scanner extracted by the simple and leaky WGP models in Section III highlight the advantage of adapting the latter. In addition, by employing two WGPs with different leakage and phase retardance parameters, the consistency of the method was verified.

Notably, we showed that using this model the extinction ratio of the imperfect polarizer used for the calibration can be simultaneously extracted using the *in situ* measurements. We showed that the calibration equations with the unknown response of the WGP was solvable using ADAM optimization in complex space. This technique provides the possibility of exploiting an *in situ* calibration for polarimetry systems and extracting the calibration parameters of the system along with the leakage and phase retardance of the WGP. this is particularly advantageous because advance knowledge of the frequency-dependent Jones matrix of the WGP used in the calibration requires separate characterization of the WGP in a separate setup. This can introduce additional sources of error due to several inaccuracies. First, the degradation of the wires can change the frequency response of a WGP to $t_{\|}$ and/or $t_{⊥}$ over time. Second, a separate transmission measurement using a separate setup might not be always available, is time consuming, or can introduce further errors in the WGP response if the transmission setup is not well-characterized. Apart from separate measurements, the use of electromagnetic models is usually complicated and might not adequately explain the leakage and phase retardance of a polarizer over a wide spectrum. Moreover, such models are based on the nominal diameter and period of the wires, which might differ from the actual values, as seen in Fig. 6. The ability to characterize an unknown polarizer along with the calibration of the system is a powerful self-calibration method for polarimetry systems.

As discussed in Section II C, the presence of the discriminant term introduces two possible solutions for the system of equations. These two solutions are hardly distinguishable in the low SNR portion of the spectrum, since the error in $A_{5}$ increases with lower SNR. This further limits the useful bandwidth of the system to only ~0.15–1.1 THz. Additionally, one key experimental consideration of the proposed calibration technique is the consistency of the phase information between measurements. Small timing jitters and long-term drift of the system due to temperature changes and non-linearity of the motion of piezo motors in the femtosecond laser cavity can limit the phase consistency of the THz-TDS system between measurements^43,66^.

One limitation of the current *in situ* calibration technique is that it assumes the frequency response of the WGPs is homogeneous. Transmission measurements were performed on the entire surface of the WGPs to verify the spatial homogeneity of the extinction ratio. In case of uneven degradation of the wires or damage to some wires of a WGP, the spatial and angular dependence should be carefully considered. In future studies, we will explore such spatial variations of the polarizer as well as incorporate polarimetric measurement capability using the proposed *in situ* calibration technique in our THz PHSR Scanner for diagnosis of burn injuries^67^ and the corneal scanner^68^ to quantify depolarization and birefringence in biological tissue.

## v. CONCLUSION

In this work, an *in situ* calibration technique for THz polarimetric imaging systems was introduced based on a rotating polarizer placed in front of a mirror. Due to the broadband nature of THz-TDP systems, the importance of incorporating a leaky model of the calibration polarizer was essential for accurate determination of the calibration parameters of the instrument. By formulating the characteristic Jones matrix of the system using an unknown and imperfect frequency response of the calibration polarizer and solving the resulting equations using optimization in complex Riemann space, both the system calibration and the spectral behavior of the polarizer were extracted simultaneously. This approach enables an *in situ* calibration configuration that improves accuracy and eliminates the need for separate polarizer characterization. The consistency and robustness of the method were verified using two different wire grid polarizers, highlighting its potential for reliable calibration of THz polarimetric measurements.

## Data Availability

The data that support the findings of this study are available from the corresponding author upon reasonable request.
